# Ultrathin sputter-deposited plasmonic silver nanostructures†

**DOI:** 10.1039/c9na00762h

**Published:** 2020-01-23

**Authors:** Selina Goetz, Martin Bauch, Theodoros Dimopoulos, Stephan Trassl

**Affiliations:** AIT Austrian Institute of Technology, Center for Energy, Photovoltaic Systems Giefinggasse 4 1210 Vienna Austria MartinBauch1@gmx.de Theodoros.Dimopoulos@ait.ac.at; HUECK FOLIEN GmbH Gewerbepark 30 4342 Baumgartenberg Austria

## Abstract

In this study, ultrathin silver plasmonic nanostructures are fabricated by sputter deposition on substrates patterned by nanoimprint lithography, without additional lift-off processes. Detailed investigation of silver growth on different substrates results in a structured, defect-free silver film with thickness down to 6 nm, deposited on a thin layer of doped zinc oxide. Variation of the aspect ratio of the nanostructure reduces grain formation at the flanks, allowing for well-separated disk and hole arrays, even though conventional magnetron sputtering is less directional than evaporation. The resulting disk–hole array features high average transmittance in the visible range of 71% and a strong plasmonic dipole resonance in the near-infrared region. It is shown that the ultrathin Ag film exhibits even lower optical losses in the NIR range compared to known bulk optical properties. The presented FDTD simulations agree well with experimental spectra and show that for defect-free, ultrathin Ag nanostructures, bulk optical properties of Ag are sufficient for a reliable simulation-based design.

## Abstract 2

A new fabrication approach allows for an easy-to-produce Ag disk–hole array showing strong plasmonic modes in experiment and simulation.

## Graphical abstract

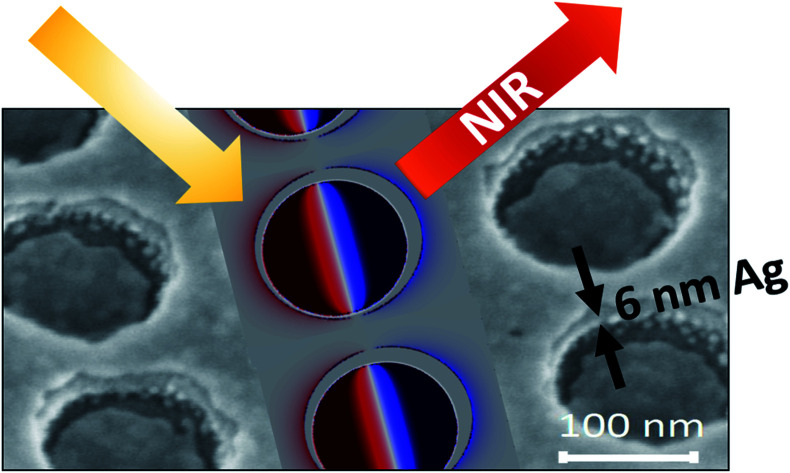

## Introduction

The scientific research in ultrathin metals is mainly driven by applications as flexible transparent electrodes, combining high transparency, low sheet resistance and mechanical stability. Typical metals employed are gold (Au), copper (Cu) and silver (Ag), which are deposited by sputtering and evaporation techniques.^1^ In terms of optical properties, Ag is considered by far the best choice owing to the low optical losses in the visible and near infrared region.^2^ However, due to the high free surface energy of silver, thin film growth starts with the nucleation of discrete silver islands,^3^ which hampers the fabrication of extremely thin continuous films. Various strategies have been developed to lower the threshold thickness for a continuous film, such as the deposition on top of dielectric buffer layers,^4^ use of metal seeding layers,^5^ surfactants^6^ and metal doping.^7^

For instance, continuous Ag layers with thickness as low as 6 nm have been realized by a partially oxidized Ag wetting layer^8^ or by oxygen doping of Ag on ZnO-coated substrates.^9^ Ag films doped with Cu showed continuous film formation at 6 nm on a glass substrate.^10^ Ag films doped with Al have been shown to form continuous 7 nm-thick films on a fused silica substrate, however optical characterization revealed significantly higher optical losses compared to pure Ag layers.^11^

The simulation-based design of nanostructures incorporating an ultrathin metal requires the precise knowledge of the optical properties of the metal. The optical properties of ultrathin Ag films can vary strongly from bulk optical properties.^12,13^ The reason for deviations is commonly found to be the non-continuity of the metal and therefore the excitation of localized surface plasmon resonances that occur at grains or in voids. In addition, surface roughness of otherwise continuous films is suggested to alter the optical properties of thin films,^14,15^ as the surface scattering of conduction electrons leads to increased losses compared to the bulk.^16^ More fundamentally, band calculations with density functional theory for thin Au films predicted a significant change of optical properties with significant anisotropy arising.^17,18^

However, the nanostructuring of ultrathin metals holds potential for transparent electrodes with high transmittance or for introduction of additional functionalities, such as surface-plasmon-induced filter properties. Examples of nanostructured ultrathin metals in the literature include a 14 nm thick Au nanohole array^19^ and a one dimensional plasmonic colour filter with 9 nm Ag on a 1 nm Al seed layer.^20^ Let us note that the two investigated geometries exhibited a significant difference between simulation and experiment. In another example, the deposition of a continuous, 8 nm-thick Ag on a periodic, nanodome polymer structure, pretreated with oxygen plasma, resulted in a transparent electrode with high transmittance, leading to increased power conversion efficiency when applied in organic solar cells.^21^

Precise patterning of periodic plasmonic nanostructures can be realized by various lithography techniques, such as electron beam lithography (EBL), focused ion beam (FIB) milling and nanoimprint lithography (NIL). EBL and FIB are very elaborate and costly fabrication techniques and are only employed for small prototype test structures, while NIL allows for large-scale realization of nanostructures. Nevertheless, NIL requires additional etching and lift-off steps when isolated periodic metallic nanoparticles are required. An alternative route for NIL-fabricated metallic nanostructures is the directional metal deposition on an NIL-structured resist without further lift-off or etching. In case the imprinted resist is a periodic disk or hole array, metal deposition results in the formation of nanodisks, separated from a perforated metal hole array by a metal-free gap. These kinds of structures have been investigated as plasmonic colour filters,^22,23^ in plasmon-enhanced fluorescence,^24^ as well as for plasmonic biosensors.^25,26^

The present work aims to combine the aforementioned aspects of simple nanostructure fabrication by NIL (without further lift-off or etching steps) with ultrathin Ag deposition to form plasmonic disk–hole arrays with high visible transmission and strong plasmonic resonance in the near-infrared range.

Therefore, thin film growth by conventional magnetron sputter deposition on different planar and structured substrate configurations is studied to obtain defect-free Ag films with thickness below 10 nm. An ultrathin disk–hole array is realized without any additional lift-off steps, enabling an easy-to-produce, highly transmissive silver plasmonic nanostructure.

Numerical simulations of planar and patterned designs are used to analyse the optical properties of ultrathin Ag and to discuss localized plasmonic modes present in the disk–hole array.

## Experimental

### Thin film deposition

All AZO (Al-doped ZnO) and Ag films are prepared using a direct current (DC) magnetron sputtering system (Leybold Univex 450C, 3SC Leybold, Germany) with a base pressure of 1.9–7.0 × 10^−8^ mbar. AZO is deposited from a 4 inch target of zinc oxide (ZnO) with 2 wt% aluminum oxide (Al_2_O_3_) in a pure argon (Ar) atmosphere at 1.0 × 10^−3^ mbar and with a sputter power of 60 W, resulting in a sputter rate of 0.294 nm s^−1^. Ag is deposited in pure Ar at a pressure of 2.0 × 10^−3^ mbar from a 4 inch target of Ag at 120 W sputter power, yielding a rate of 1.809 nm s^−1^.

### Fabrication of the nanostructures

The nanostructures were realized by nanoimprint lithography. A silicon (Si) master structure was obtained from EULITHA AG (Switzerland) having periodic pillars with 90 nm in height, 120 nm in diameter and a period of 200 nm. The master was silanized with trichloro(1*H*,1*H*,2*H*,2*H*-perfluorooctyl)silane (448931, Sigma-Aldrich, Austria) to reduce adhesion of the NIL-resist and ensure complete delamination. Subsequently, a drop of 1 μl of UV-resist (OrmoStamp®, Micro Resist Technology GmbH, Germany) was placed on the master and a cleaned soda-lime glass substrate (25 mm × 25 mm) was placed on top without applying additional pressure. UV-curing of the resist was done with a UV lamp (Polylux500, Dreve Optoplastik GmbH, Germany) with a wavelength range of 315–400 nm for 5 min, followed by a 30 min hard-bake on a hotplate at 150 °C. The resulting structure is an array of cylindrical holes. Afterwards the structure was gradually altered in height and diameter using Ar ion beam etching (IBE) (IonSys 500, Roth & Rau AG, Germany) at a beam-to-substrate-angle of 40° for different etching times of 0 min to 3 min (at 500 V accelerator voltage and microwave power of 375 W). Subsequent thin film sputter deposition of AZO and Ag (as described before) resulted in the formation of a disk–hole array without post-deposition lift-off or etching steps.

### Morphological and optical characterization

The nanostructures were imaged with an atomic force microscope (AFM) (Molecular Imaging PicoPlus) in tapping mode, using SSS-NCHR-10 (Nano world AG) tips. The surface morphology and metal growth were studied using scanning electron microscopy (SEM) (Zeiss, SUPRA 40) at 5 kV acceleration voltage with an in-lens detector. Optical transmittance and reflectance spectra were obtained using a Fourier transform infrared spectrometer (FTIR) (Vertex 70, Bruker Corporation), equipped with an additional visible light source. All measurements were performed with unpolarized light. Transmittance was measured at normal incidence and referenced to air, while reflectance was determined at an incidence angle of 13° from the coated side of the sample and referenced to a calibrated mirror (STAN-SSH-NIST, Ocean Optics).

### Simulation

Simulations of planar thin films were performed by the transfer matrix method (TMM).^27,28^ The complex refractive index of AZO was taken from the literature.^29^ For the Ag thin film, data from three frequently used sources, namely Palik,^30^ Johnson and Christy^31^ and Rakic *et al.*,^32^ were compared with experimental values to find the best fitting data for the complex refractive index.

TMM simulations of transmittance were performed at normal incident light, while reflectance was calculated at 13° to match experimental conditions. The complex permittivity of 6 nm thick Ag film on 10 nm thick AZO film was retrieved from transmittance and reflectance measurements in combination with a TMM algorithm.^33,34^

Simulations of periodic nanostructures were performed by the finite-difference time-domain (FDTD) method (FDTD Solutions from Lumerical Solutions, Inc., Canada). A single unit cell of the nanostructure with periodic boundary conditions in the *x*- and *y*-direction and perfectly matched layers (PMLs) in the *z*-direction was used for simulation of transmittance and reflectance for normal incident light. The unit cell height was fixed to 500 nm in the *z*-direction and 16 layers of PMLs were used. A broadband plane wave light source was used and is incident from the air-side. The optical properties of AZO were taken from the literature^29^ and the complex permittivity of Ag was taken from Rakic *et al.*^32^ The glass/OrmoStamp substrate was assumed with constant refractive index *n* = 1.516 over the complete spectral range and considered infinite in the minus *z*-direction. The surface charge density at the Ag/air interface was calculated from the divergence of the electric field at the respective spectral resonance position. A uniform 1 nm-mesh in the *x*-, *y*- and *z*-direction was employed for all simulations. Mesh convergence was checked by an additional simulation with a 0.5 nm uniform mesh, where no additional improvement of the spectrum was achieved. For simulation of angle-dependent transmittance for different polarizations, periodic boundary conditions in the *x*-direction were replaced by Bloch-periodic boundary conditions.

The uncoated air/glass interface, which is otherwise not considered in the TMM and FDTD simulations, due to practical reasons, was taken into account using simple analytical equations.^15^ This allows for a direct comparison between experiment and simulation.

## Results and discussion

The realization of ultrathin films is often restricted by dewetting of the deposited films. Due to the high free surface energy of silver, thin film growth starts with the nucleation of discrete silver islands followed by cluster coalescence until the percolation threshold is reached and finally a continuous film is formed.^35^ The progress of nucleation, island growth and coalescence strongly depends on the surface and interface energy of the substrate affecting the wetting properties, adhesion strength and cluster mobility of the adsorbed silver.^3^ With decreasing energy difference between the Ag and the substrate, the Ag atoms become more strongly bound to the substrate than to each other, yielding enhanced wetting properties and a more stable film.^35^ Since the morphology of the metal film strongly influences the optical properties,^10,36^ an investigation of Ag growth on different planar substrate configurations, namely soda-lime glass, NIL-resist (OrmoStamp) and 10 nm AZO layer, is performed prior to implementation in nanostructures.

**Fig. 1:**
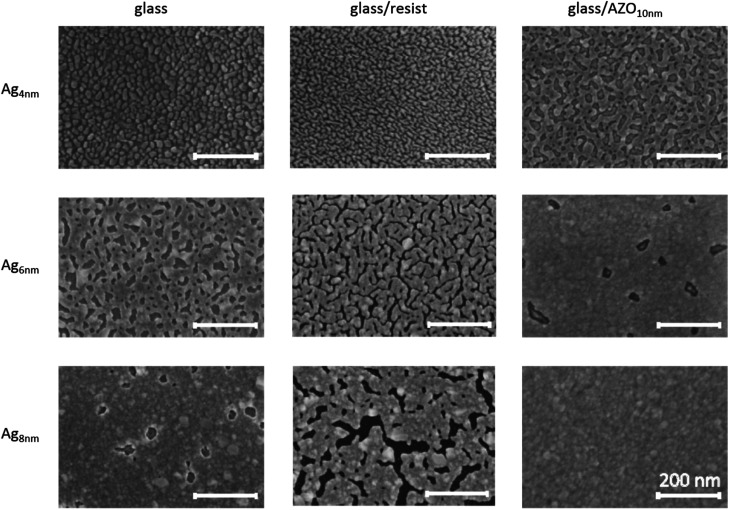
SEM characterization of ultrathin sputter deposited Ag films with increasing thickness (4 nm, 6 nm and 8 nm) on different substrate materials. The investigated substrates are bare glass, NIL-resist (OrmoStamp) and 10 nm AZO on glass, respectively. The scale bar is 200 nm in all SEM images.

In Fig. 1, the evolution of ultrathin Ag layers on these substrates is presented. While the deposition on bare glass offers a comparison with other studies,^4^ the Ag growth on the planar NIL-resist (OrmoStamp) is essential to investigate for the following nanostructuring process. ZnO has been used as a supporting material for improved wetting,^37–39^ hence the introduction of the AZO layer is expected to change the adhesion properties of Ag while maintaining the transparency of the substrate. All Ag layers are deposited under the same sputter conditions, where a high sputter power of 120 W was chosen to support continuous film growth even at low thicknesses.^40^ It should be noted that there is no difference in Ag growth irrespective of whether the AZO layer was deposited on the planar resist or directly on glass (see Fig. S1†).

4 nm Ag samples on glass and resist show small isolated islands, while the Ag film on AZO is already coalesced. The increased wettability of AZO with respect to glass cannot be explained by higher substrate surface energy (for contact angle measurement data see Table S1 in the ESI†). Instead, the chemical bonding of Ag atoms to the underlying AZO surface as well as a beneficial crystallographic arrangement is considered as a contributing factor for the improvement in Ag wetting.^35^ The polycrystalline AZO film with a prominent (002) wurtzite peak and a weaker (013) peak in the XRD pattern (see Fig. S2†) may promote the stable arrangement of Ag with (111) and (202) orientation, compared to the amorphous glass substrate. When increasing the Ag thickness to 6 nm, the islands on glass and the resist start to coalesce, whereas on AZO the film is already continuous, exhibiting only a few occasional holes. Further increase of the Ag thickness to 8 nm yields a defect-free film on AZO and a film with sporadic holes on glass that is similar to the 6 nm Ag layer on AZO. The 8 nm thick Ag layer on the resist has enlarged meandering gaps compared to 6 nm Ag, showing the tendency of Ag migration and high surface mobility. Therefore, Ag adatoms on the resist coalesce with each other rather than sticking on the substrate and consequently the filling of voids is delayed. This behaviour is confirmed by contact angle measurements, where the planar resist exhibits a significantly higher contact angle (CA) than AZO or glass (see Table S1†). The significantly improved adhesion of Ag on AZO, compared to its deposition on the resist, makes AZO a necessary base layer for the realization of an ultrathin Ag film on nanostructures.

**Fig. 2:**
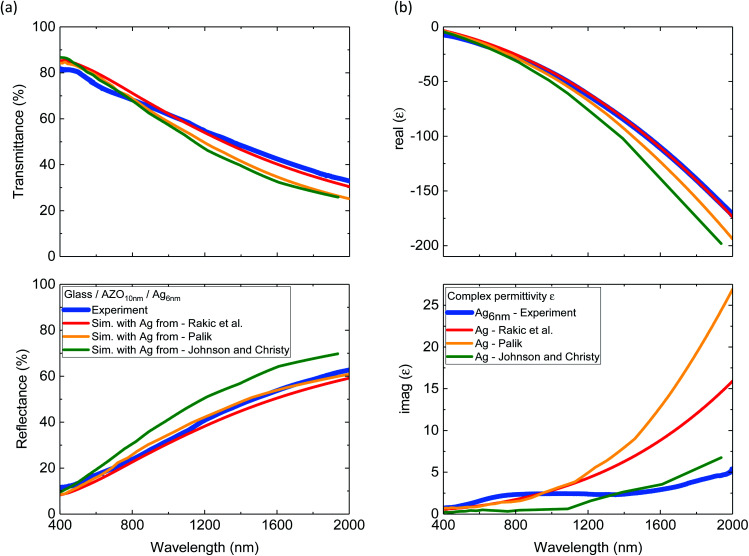
(a) Experimental and simulated (Sim.) transmittance and reflectance spectra of 6 nm Ag on 10 nm AZO. Simulations are performed for continuous layers with different literature values of bulk Ag. (b) Real and imaginary part of complex permittivity of 6 nm Ag derived from the experiment and values from the literature.

Experimental transmittance and reflectance spectra of 6 nm Ag on top of 10 nm AZO layer are shown in Fig. 2(a). TMM simulations of the layer system were performed with 3 frequently used Ag dispersion relations from the literature, while the optical properties of AZO were taken from a previous study.^29^ Even though the ultrathin Ag layer has occasional holes, which are not considered in the simulation, a good agreement with simulation and experiment is observed. The good agreement motivated us to calculate the complex electrical permittivity, despite the defects in the film. The comparison of the derived complex permittivity of the 6 nm Ag and the literature values is given in Fig. 2(b). The real part of the experimental permittivity agrees best with the Ag permittivity from Rakic *et al.* The experimentally derived imaginary part of permittivity shows a different trend compared to the literature with higher values below *λ* = 1000 nm and significantly lower values above *λ* = 1000 nm compared to those reported by Palik and Rakic *et al.* The imaginary part of the permittivity is associated with the absorption losses in a material.^2,41^ Especially the lower optical losses above 1000 nm compared to those reported by Palik and Rakic *et al.* show great promise for plasmonic applications in the near-infrared with high plasmonic quality factors.^2^ To our knowledge no other study has shown so good agreement between ultrathin Ag film below 10 nm and the bulk optical properties.

**Fig. 3:**
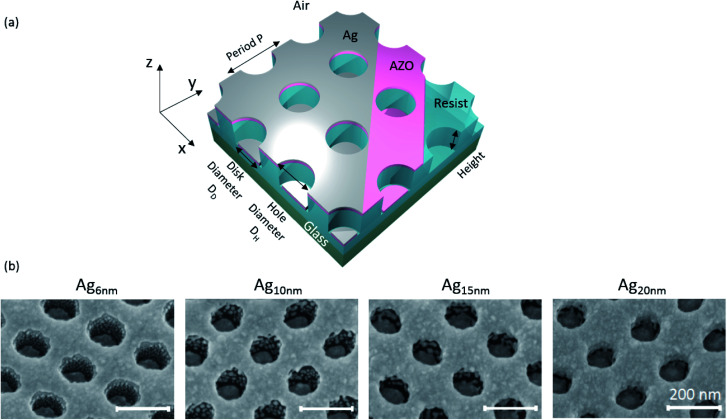
(a) Schematic of the nanostructure under investigation. (b) SEM images (at 30°-tilted view) for different Ag thicknesses on 10 nm AZO deposited on a nanostructured resist (height 90 nm, period 200 nm and diameter app. 100 nm). The evolution of grain growth on the sidewalls during sputter deposition can be observed. The scalebar is the same for all images.

Fig. 3(a) schematically presents the configuration of the nanostructures investigated in the following, where the NIL-resist is patterned with cylindrical holes and AZO and Ag are subsequently deposited. The sketch represents an ideal situation, where no material is adsorbed on the sidewalls, resulting in a perforated Ag film raised above an array of Ag disks. Directional metal deposition techniques, typically applied for such nanostructures without a lift-off step, are thermal- and e-gun evaporation in ultrahigh vacuum. Nevertheless, even directional deposition techniques cannot avoid the formation of metallic grains on geometrically shadowed regions due to metal diffusion from adjacent layers.^24,42,43^ These grains can interact with local electric fields of excited surface plasmons and alter the optical properties of plasmonic structures. Consequently, detailed investigation of grain formation is important. In contrast to highly directional deposition techniques, magnetron sputtering exhibits a cosine emission profile and higher collision rates in the gas phase.^44^ Therefore the incident Ag particles have a noticeable velocity component parallel to the surface and are thus able to directly adsorb on the sidewalls. However, the narrow holes lead to shadowing effects reducing the deposition rate on the sidewalls as well as on the bottom disks. Fig. 3(b) shows SEM-images presenting the Ag growth on structured substrates with a 10 nm AZO base layer and varying Ag thicknesses. At 6 nm thickness the deposited material on the sidewalls grows as small, separated grains, similar to the film growth on planar substrates at very small thicknesses. There is a clear separation of the disk array and the hole array that originates from substantial shadowing, which amplifies perturbations in the surface^45^ as well as surface energy minimization by dewetting the corners. For increasing Ag thickness, the grain size on the sidewalls increases continuously. While the disks are still completely separated at 10 nm Ag thickness, the grains start to connect with the disks on the bottom, as well as with the hole array at 15 nm Ag thickness. Further increase to 20 nm Ag results in almost complete step-coverage and hence electrical connection of the disks and holes. Consequently, the excitation of localized surface plasmons in the disks and holes will be less pronounced. Therefore, a small Ag thickness is not only beneficial for high optical transparency but also essential for the exploitation of localized plasmonic resonances, ensuring the separation of disk and hole arrays.

Another interesting feature arises from the comparison of the structured AZO_10nm_/Ag_6nm_ sample with the same layer configuration on a planar substrate (see Fig. 1(b)). While the planar 6 nm Ag film is still disrupted by occasional holes, the perforated hole array film, as well as the disks themselves is free of these defects (see Fig. S3†). It is noted here that the holes appear on the planar substrate regardless of whether the deposition is done on plain glass or on glass covered with the resist (Fig. S1†). Although contact angle measurement data (see Table S1†) show different wetting properties for the planar and structured samples, it cannot explain the improved Ag growth on the structured sample. The higher contact angles on the structured configurations probably come from macroscopic dewetting effects due to the nanostructured surface, where the drop does not cover the same effective surface area as in the planar configuration. Another possibility is that the distance that Ag adatoms (or clusters) may diffuse is modified by the spatial restrictions posed by the nanostructured substrate, with a smaller diffusion length leading to faster island percolation. The reported large diffusivity of Ag on various weakly interacting substrates supports this argument.^46,47^ In any case, it seems that the silver growth on AZO is improved by the presence of the structure, making stable nanostructured Ag films as thin as 6 nm possible.

**Fig. 4:**
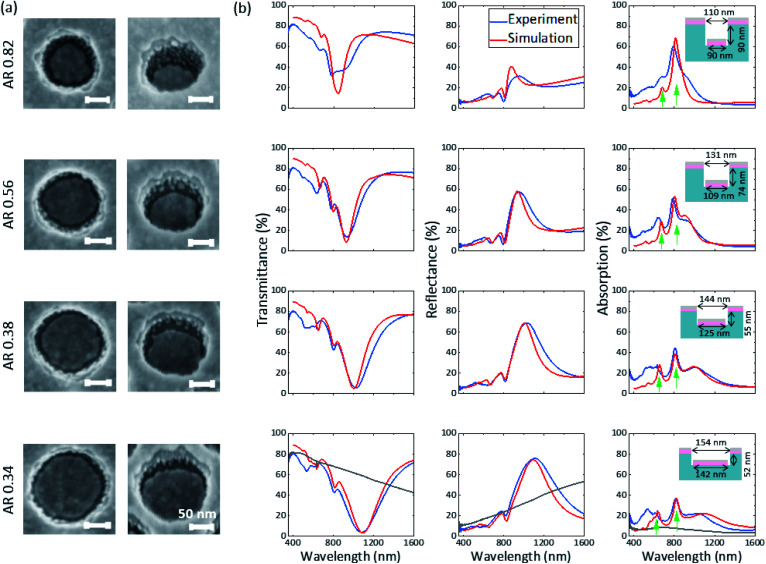
(a) SEM images at normal and 30° tilted view for different aspect ratios (ARs) of the disk–hole array with 10 nm AZO and 6 nm Ag. (b) Experimental (blue) and FDTD-simulated (red) transmittance, reflectance and absorption spectra of the corresponding structures. Geometry parameters used for the simulations are indicated by the schematic insets. The grey solid line in the last row represents the experimental spectra of the planar structure with same layer thicknesses.

The formation of Ag grains on the side walls of the nanostructures was further investigated by change of the geometry of the nanostructured resist, prior to deposition of 10 nm AZO and 6 nm Ag. The geometry parameters of the nanostructured resist were changed by IBE with different exposure times (see Fig. 4 with 0 min, 1 min, 2 min and 3 min etching time from the top to bottom row, respectively). The beam-to-substrate angle is chosen in a way that the diameter and the height of the cylindrical holes are increased and decreased, respectively.

Fig. 4(a) shows the SEM images of the coated structure with different aspect ratios (AR = height/hole diameter) for different etching times. With decreasing aspect ratio, the number of grains is significantly reduced while the separation of disks and holes is clearly maintained or even improved. At the lowest aspect ratio nearly all grains are merged with the upper rim of the hole array. The corresponding experimental optical spectra are shown in Fig. 4(b) and are compared to FDTD simulations (with Ag bulk properties taken from Rakic *et al.*) of a disk–hole array without consideration of the grains. The geometrical parameters for the simulation are deduced from SEM normal view images (disk diameter and hole diameter) and from AFM measurements (height of the nanostructure, data not shown) and are indicated in the insets. The spectra show a multitude of optical modes which manifest as dips in transmittance and peaks in reflectance and absorption. The comparison of simulated and experimental spectra reveals that with decreasing AR a better agreement between experiment and simulation is achieved. A possible explanation for this behaviour is the drastically reduced number of grains for lower ARs, which are not accounted for in the simulations. Especially at the highest AR of 0.82 the deviation between simulation and experiment is significant in the complete wavelength range, while for the disk–hole array with low AR of 0.34 the optical modes in the near-infrared region agree well in spectral position and magnitude. A possibly reduced disk thickness in the hole due to the shadowing effect in the high AR case (AR 0.82) has been ruled out as explanation for the strong deviation by additional simulations (see Fig. S4†). All samples exhibit lower experimental transmittance and higher absorption in the visible range compared to simulation. This can be explained by the higher imaginary part of the complex permittivity in the visible range as discussed earlier. The low optical losses of the ultrathin Ag in the NIR, already discussed for the planar case, can be especially well observed in the absorption spectra of the AR 0.34 structure above 1050 nm, where the absorption spectrum of the experiment is lower than the simulation employing bulk optical properties.

The most prominent optical mode in the NIR shows a spectral dependence on the change of geometry parameters, while two optical modes well distinguished in the absorption spectra and marked with two green arrows are surprisingly stable towards change of parameters.

In the last row, the spectra of the planar structure (grey line) with same layer thicknesses of AZO and Ag are additionally shown. Comparing average transmittance in the visible range (here considered from 380 nm to 700 nm) yields 71% for the disk–hole array and 77% for the planar layers. Above 700 nm the transmittance of the disk–hole array drops significantly faster compared to the planar layer. At the main resonance at around 1100 nm, experimental transmittance drops as low as 4% for the disk–hole array, while the planar counterpart shows a transmittance of 59%. Above 1360 nm the transmittance of the disk–hole array rises above the transmittance of the planar structure. Considering that the solar spectrum above this wavelength contributes only app. 11% to the total solar intensity (see the ESI of Bauch *et al.*^29^), these properties qualify the presented structure with an aspect ratio of 0.34 as an easy-to-produce filter to block solar radiation in the NIR. Therefore, the aging of the ultrathin silver film (for AR 0.38) when exposed to air was observed over several weeks, showing the known weakening and red-shift of the resonant transmittance dip (see Fig. S5†).^48^ However, the tarnishing of the silver and with it the reduction of optical performance happened much slower than expected from other studies.^11,49,50^ In the first week after deposition no significant change was observed. After 4 weeks of air exposure, the transmittance minimum of the strong NIR-mode shifted by 40 nm to higher wavelengths and weakened about a total of 2% compared to the as-deposited measurements.

**Fig. 5:**
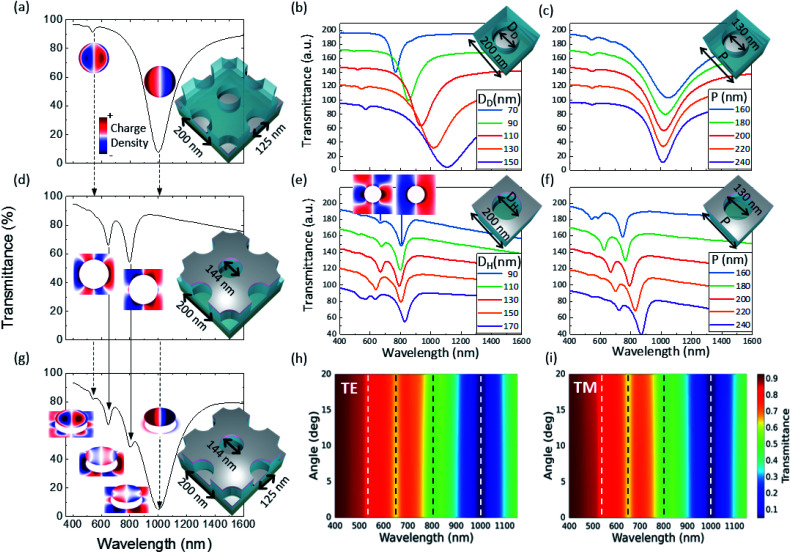
Simulated transmittance spectra and surface charge density at resonance positions for the periodic (a–c) disk array, (d–f) hole array and (g) disk–hole array for a variety of geometrical parameters (given in the insets). An offset has been introduced in the transmittance parameter variations for better distinction. Scale of charge density is adapted for every resonance. Angle wavelength map for the disk–hole array (with same parameters as in (g)) for (h) TE-polarization and (i) TM Polarization. Resonance positions are indicated by dashed lines. All simulations are done for resist height 55 nm, AZO thickness of 10 nm and Ag thickness of 6 nm.

A detailed analysis of the optical modes present in the disk–hole array is shown in Fig. 5. The disk–hole array is decomposed into its main components, a disk array and a hole array, to facilitate mode analysis and investigate possible coupling between disks and holes. The transmittance spectra and charge density maps of the disk array are shown in Fig. 5(a). A strong plasmonic dipole resonance is observed in the near-infrared region around *λ* = 1000 nm and a much weaker multipole excitation in the visible range. Multipole excitations in nanoparticles have been frequently observed for a variety of geometries including metallic nanodisks.^51–53^ An increase in disk diameter (see Fig. 5(b)) results in a typical red shift of the localized surface plasmon resonance of nanodisks.^22,51,54^ The change of disk array period (see Fig. 5(c)) has only a minor influence on the dipole resonance position. With decreasing period, a slight red shift is observed due to near-field interaction between the disks.^55^

The transmittance spectrum and charge density of the hole array can be seen in Fig. 5(d). Two multipole hole array modes^56^ can be observed. These modes are of localized nature as the change of incident angle for TE and TM polarization does not influence resonance position (data not shown). Furthermore, the localized nature of the hole array modes has been investigated in one of our previous studies.^29^ Interestingly, a dipole resonance in the hole array, as observed by other authors,^23,56^ is not readily seen. Only a decrease in hole diameter (see charge distribution in Fig. 5(e)) reveals a clear dipole hole mode. It seems that the formation of a clear dipole mode in the hole array is inhibited when the hole diameter becomes large with respect to the hole array period.

In contrast to the disk array, the spectral position of the hole array resonances is only weakly influenced by a change in diameter. This explains the stable resonance positions of the hole array modes observed in Fig. 4 (green arrows) with a fixed period. The hole array resonances are strongly influenced by the period of the array, even for a non-diffractive array, as reported by Parsons *et al.*^57^

The simulation of the disk–hole array in Fig. 5(g) (same parameters as the experimental structure in Fig. 4 with AR 0.38) reveals no significant coupling between the disk array and hole array as resonance positions compared to the individual components do not exhibit a spectral shift. The charge density of the disk–hole array shows same charge distribution as the individual components with the respective counterpart showing the induced image charges.^58^ The angle wavelength maps for transverse electric (TE) and transverse magnetic (TM) polarization of the disk–hole array (see Fig. 5(h) and (i)) show angle independent modes and therefore confirm the localized nature of all observed plasmon modes.^57^

## Conclusion

This study reports ultrathin, Ag-based plasmonic nanostructures, fabricated by nanoimprint lithography and DC magnetron sputtering without lift-off steps. With its ease of fabrication, this process can be easily adapted for high-throughput and large-scale roll-to-plate or roll-to-roll production for rigid or flexible substrates. Indeed, each processing step (application of the resist, nanostructuring by the mold roller, UV-curing of the resist, sputter deposition, *etc.*) can be scaled up for large surface areas. While 6 nm Ag on a planar, ZnO-based substrate configuration yielded a continuous film with occasional holes, the deposition of the same film on a nanostructure resulted in a defect-free Ag film. The comparison of optical properties of the flat 6 nm Ag with Ag bulk optical properties from the literature yielded a good agreement with slightly increased optical losses in the visible range, while losses in the NIR region were reduced. These results hold great potential for plasmonic applications of ultrathin Ag films with high quality factors in the NIR region. The comparison of experimental optical spectra of the nanostructure and FDTD simulations demonstrated likewise a good agreement, when grain formation at the nanostructure flanks can be avoided. It is shown that the bulk optical properties of Ag are in first approximation sufficient to predict optical properties of nanostructures as thin as 6 nm, when a continuous film can be reached. Therefore, the results indicate that ultrathin Ag nanostructures with high quality factors in the NIR can be achieved and reliably designed with simulation tools. The presented nanostructures show a high transmittance of 71% in the visible range due to the low Ag thickness, while a strong plasmon resonance with increased reflectance in the NIR is observed. This behaviour allows for possible application of the ultrathin Ag nanostructure as a solar control coating or an NIR-filter.

## Conflicts of interest

There are no conflicts to declare.

## Supplementary Material

NA-002-C9NA00762H-s001
