# Supplementary material for: Ultrathin sputter-deposited plasmonic silver nanostructures
Source: Nanoscale Adv. 2020 Jan 23;2(2):869–77. doi: 10.1039/c9na00762h (PMC9418784; doi:10.1039/c9na00762h)
Supplement: NA-002-C9NA00762H-s001 [file NA-002-C9NA00762H-s001.pdf]

Electronic Supplementary Material (ESI) for Nanoscale Advances.
This journal is © The Royal Society of Chemistry 2020

Supporting Information

Ultrathin sputter-deposited plasmonic silver nanostructures

Selina Goetz,[a] Martin Bauch,[a] Theodoros Dimopoulos*[a] and Stephan Trassl[b]

[a.] *Photovoltaics Systems, AIT-Austrian Institute of Technology, Giefinggasse 4, 1210 Vienna, Austria.*
* *E-mail: MartinBauch1@gmx.de; Theodoros.Dimopoulos@ait.ac.at*

[b.] *HUECK FOLIEN GmbH, Gewerbepark 30, 4342 Baumgartenberg, Austria.*

1. Ag film growth on AZO with different substrate configurations
2. Contact angle measurement
3. XRD
4. Comparison of planar and structured Ag film
5. Optical Spectra for reduced disk thickness
6. Aging of the ultrathin Ag film

1. Ag film growth on AZO with different substrate configurations

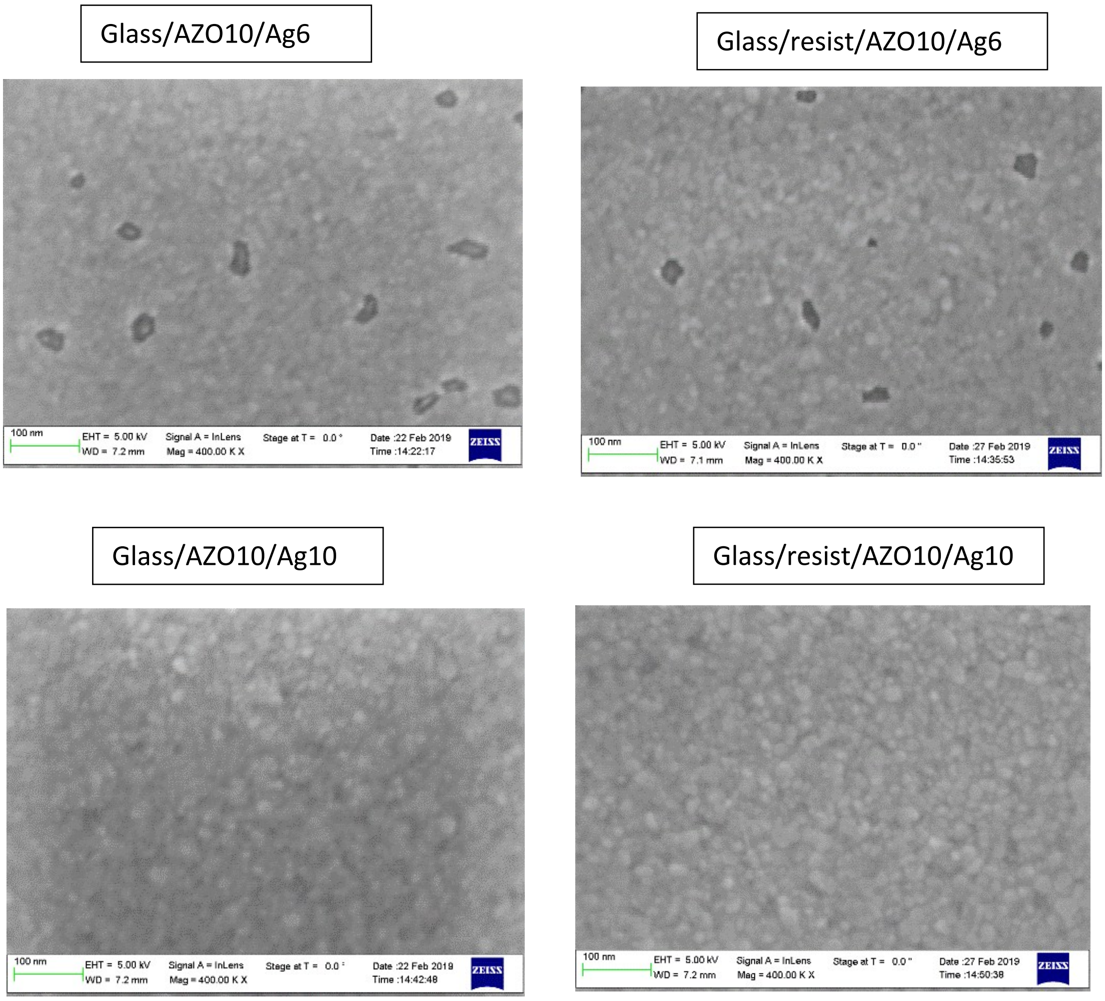

Figure S1. SEM images of AZO_{10}/Ag layers deposited on glass and on planar resist (OrmoStamp).

2. Contact angle (CA) measurement

Sample	CA with water	CA with diiodo-methane	Surface free energy
AZO on glass	27.06 (±0.32)°	41.22 (±0.84)°	69.83 ±0.72 mN/m
AZO on planar OrmoStamp	24.38 (±0.95)°	41.84 (±0.46)°	70.86 ±0.66 mN/m
AZO on structure	46.99 (±0.27)°	65.36 (±0.36)°	53.30 ±0.44 mN/m
Clean soda lime glass	19.34 (±0.27)°	48.08 (±0.34)°	71.58 ±0.35 mN/m
Planar OrmoStamp	69.79 (±0.47)°	45.50 (±0.67)°	45.12 ±0.61 mN/m
Structured OrmoStamp	67.85 (±5.87)°	53.78 (±3.30)°	43.11 ±5.16 mN/m

Table S1. Contact angle measurement data acquired by KRÜSS ADVANCE software in double sessile drop mode by an KRÜSS DSA25E using water and diiodo-methane drops in air. The surface free energy is calculated from Young's equation $\sigma_s = \sigma_{ls} + \sigma_l \cos\theta$ (https://www.kruss-scientific.com/ru/services/education-theory/glossary/surface-free-energy/, 15.07.2019).

3. X-Ray Diffractometry (XRD)

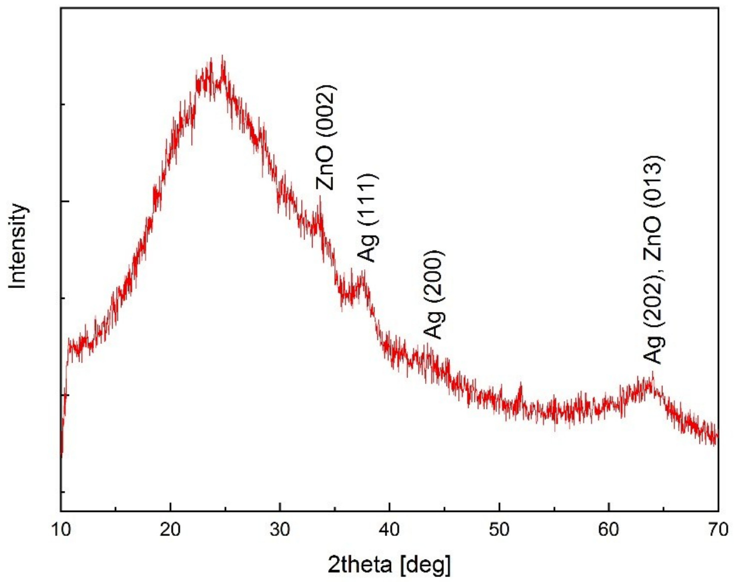

Figure S2. XRD pattern (ThermoFisher Scientific ARL Equinox 100, using a Cu-Kα source at 1.5419 Å) of planar AZO_{10}/Ag_6 on glass. The angle of incidence was set to 1 degree to gain more pronounced peaks of the thin film configuration. The most prominent peak is assigned to Ag (111), while the AZO preferentially grows in a wurtzite (002) orientation.

4. Comparison of planar and structured Ag film

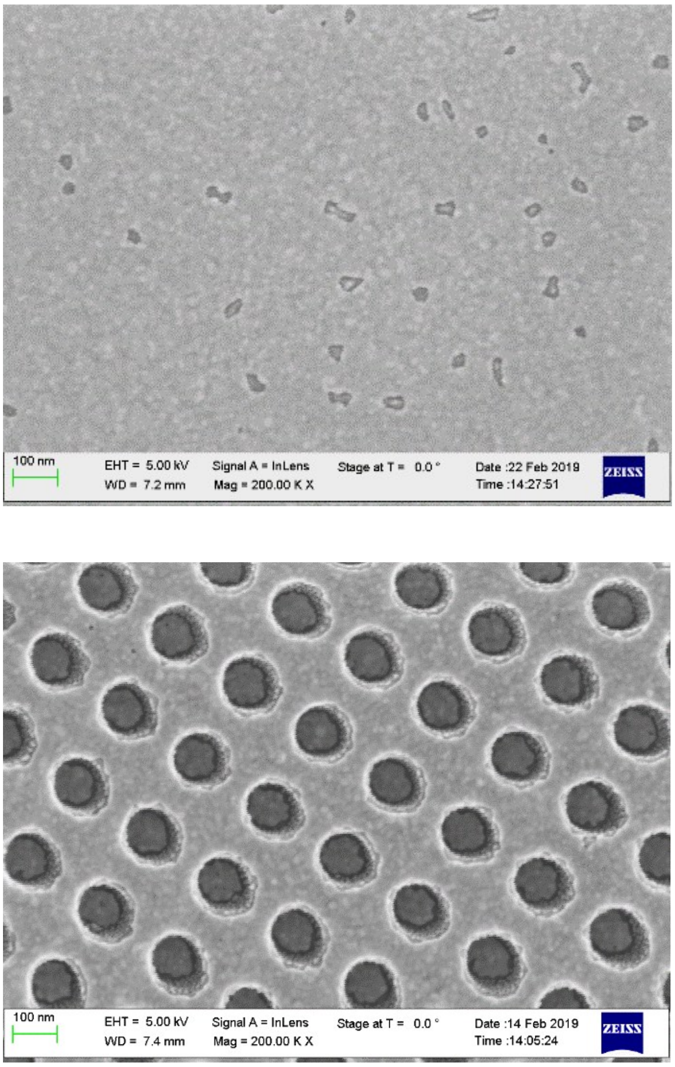

Figure S3. SEM images of a 6 nm thick Ag film on AZO on a planar (left) and structured (right) substrate at same magnification.

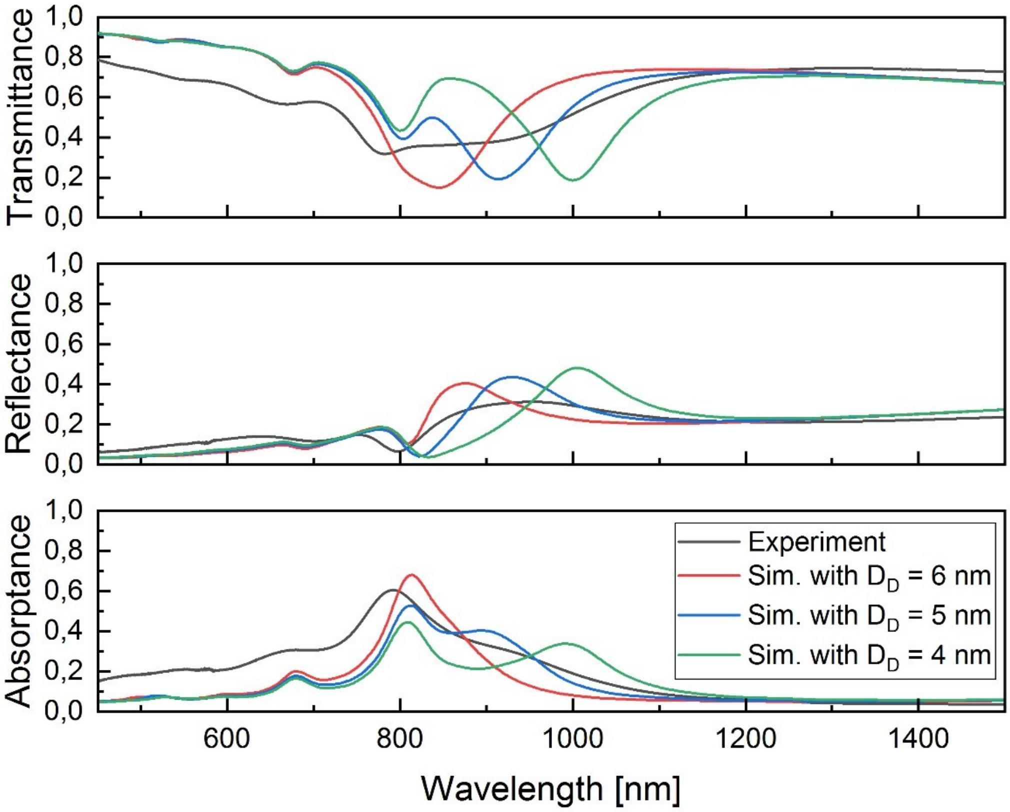

5. Optical Spectra for reduced disk thickness

Figure S4. Variation of the disk thickness in the FDTD simulation for aspect ratio AR = 0.82. While the geometry and the Ag film thickness of the hole array is kept constant at 6 nm in the simulation, the Ag disk thickness is stepwise reduced.

6. Aging of the ultrathin Ag film

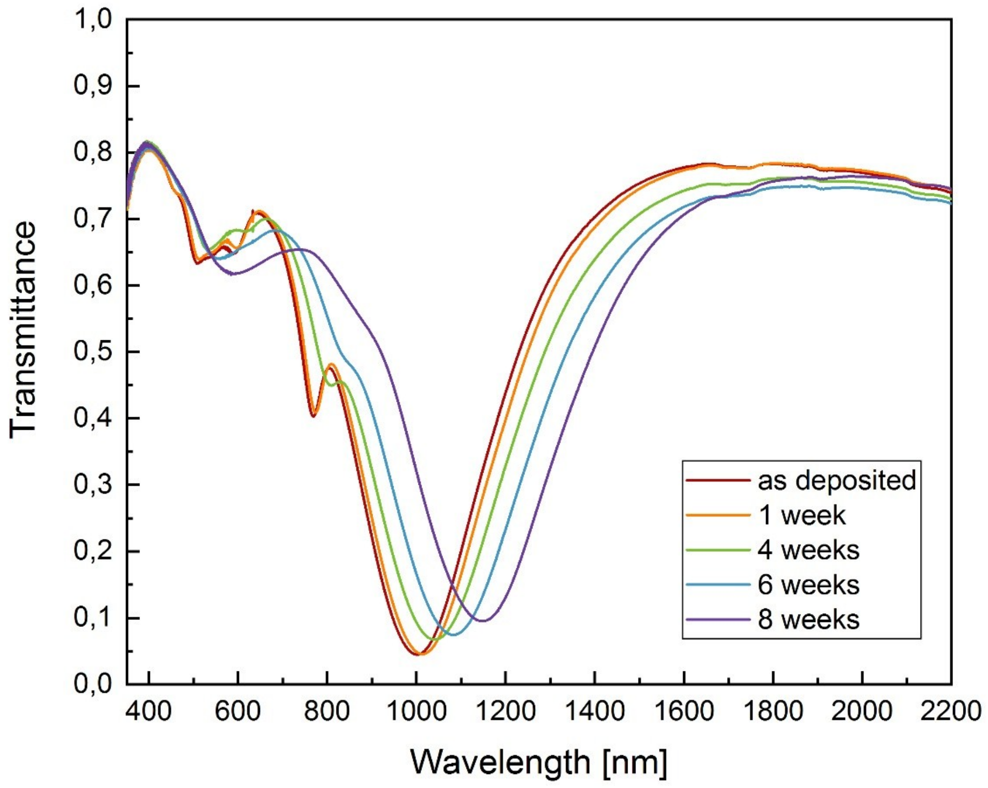

Figure S5. Change of transmittance spectrum of a 6 nm Ag film upon exposure to air. Spectra of the same film are measured after several time periods.
